# Supplementary material for: 5-Fluorouracil Conversion Pathway Mutations in Gastric Cancer
Source: Biology (Basel). 2020 Sep 2;9(9):265. doi: 10.3390/biology9090265 (PMC7563957; doi:10.3390/biology9090265)
Supplement: Supplementary file 1 [file biology-09-00265-s001.pdf]

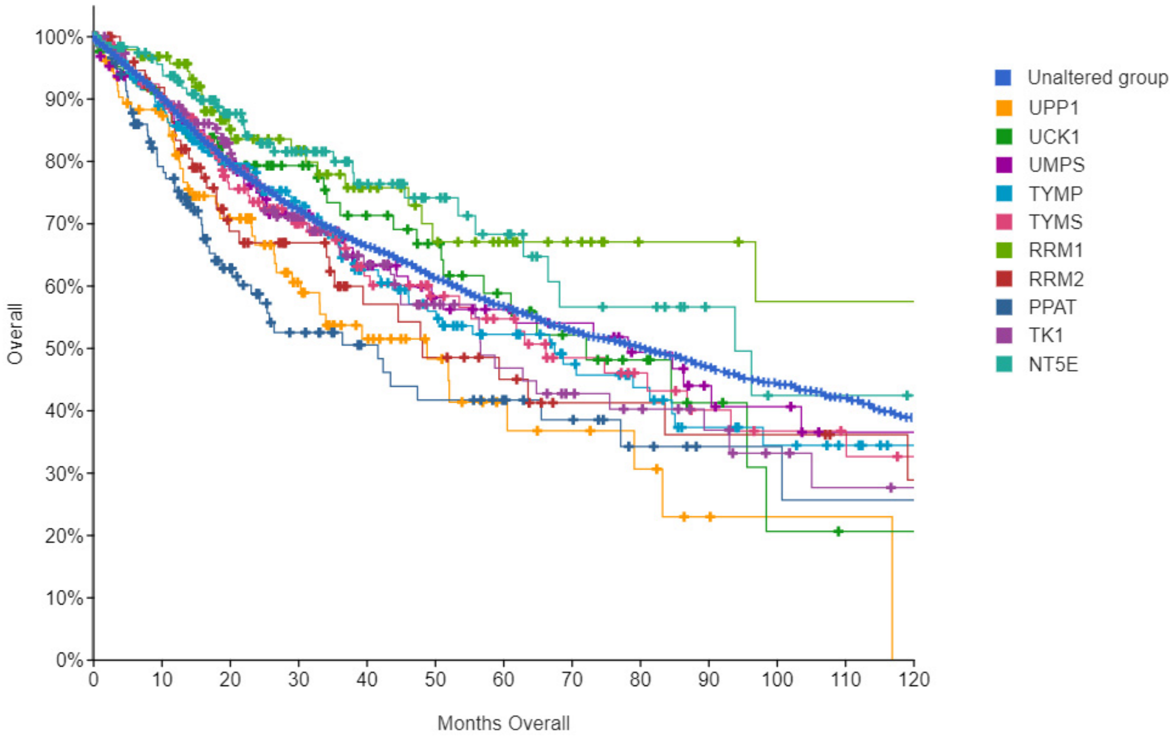

Supplementary Figure S1. Blue: unaltered group; the altered group was divided accordingly to the single mutation pattern; data retrieved from 32 studies including 10953 patients, TCGA PanCancer Atlas. Total number of patients included in each cohort: unaltered group 9467, UPP1 156, UCK1 125, UMPS 196, TYMP 262, TYMS 187, RRM1 150, RRM2 135, PPAT 195, TK1 211 and NT5E 183. Overlapping patients were excluded from the analysis.

Supplementary Table S1. The relative *p* values, calculated singularly respect the unaltered group by long-rank test, and the median month overall were reported in the table below.

Cohort	Median Month Overalls	*p* value
Unaltered group	80.74	
UPP1	48.76	3.107e-4
UCK1	88.57	0.513
UMPS	78.18	0.501
TYMP	67.46	0.190
TYMS	61.84	0.0415
RRM1	151.26	0.0176
RRM2	59.31	0.596
PPAT	77.10	0.173
TK1	58.09	0.276
NT5E	102.11	8.637e-3

Supplementary Table S2. We analyzed the co-occurrence frequency of 5-FU pathway-related mutation genes in the altered cohort, comprising 6 studies including 1365 GC patients.

A	B	A Not B	B Not A	Both
UCK1	*UMPS*	29	12	6
TYMS	*RRM1*	10	12	4
UCK1	*PPAT*	30	23	5
UMPS	*RRM1*	15	13	3
RRM1	*RRM2*	13	15	3
TYMP	*NT5E*	31	26	5
UMPS	*RRM2*	15	15	3
TK1	*NT5E*	13	28	3
UMPS	*PPAT*	15	25	3
UPP1	*TK1*	29	13	3
UCK1	*RRM1*	32	13	3
TYMP	*TK1*	33	13	3
UCK1	*RRM2*	32	15	3
UPP1	*TYMS*	30	12	2
UPP1	*TYMP*	29	33	3
UMPS	*NT5E*	16	29	2
UPP1	*RRM2*	30	16	2
UCK1	*NT5E*	33	29	2
UCK1	*TYMP*	33	34	2

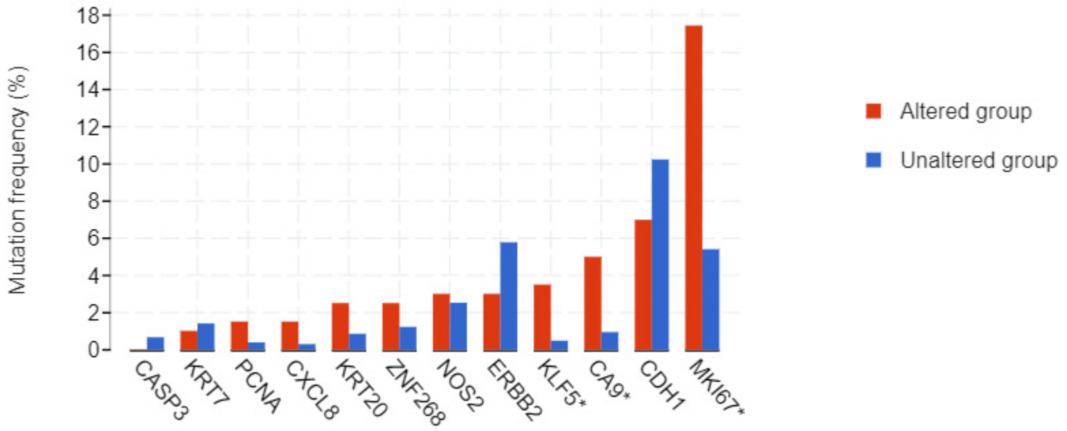

Supplementary Figure S2. The analysis of the frequency of the mutations of the most common GC- and inflammation-associated markers (CK7/20, PCNA, Ki67, EGR1, ERBB2, KLF5, CAIX, E-cadherin, IL8, NOS2 and CASP3), in the altered and unaltered groups. Briefly, the altered one includes all the patients with at least one of the 5FU metabolic genes mutated and the other one including all the "unaltered"; * $p < 0.05$.
